# Predictive biomarkers for regression in women undergoing active surveillance for cervical intraepithelial neoplasia grade 2: A prospective multicenter study in Italy

**DOI:** 10.1002/ijc.70104

**Published:** 2025-08-30

**Authors:** Helena Frayle, Silvia Gori, Alessio Pagan, Marika Soldà, Cesare Romagnolo, Egle Insacco, Licia Laurino, Mario Matteucci, Giuseppe Sordi, Enrico Busato, Manuel Zorzi, Tiziano Maggino, Annarosa Del Mistro, CIN2 Study Working Group, Annarosa Del Mistro, Helena Frayle, Silvia Gori, Rossana Trevisan, Alessio Pagan, Justyna Wojciechowska, Enrico Busato, Tiziano Maggino, Marika Soldà, Cesare Romagnolo, Licia Laurino, Pamela Zambenedetti, Egle Insacco, Mario Matteucci, Maria Teresa Gervasi, Giuseppe Sordi, Marco Torrazzina, Manuel Zorzi, Daria Minucci

**Affiliations:** ^1^ Immunology and Diagnostic Molecular Oncology Unit Veneto Institute of Oncology IOV‐IRCCS Padua Italy; ^2^ Ospedale Ca' Foncello, Local Health Unit Marca Trevigiana Treviso Italy; ^3^ Ospedale dell'Angelo, Local Health Unit Serenissima Venezia Italy; ^4^ Obstetrics and Gynaecology, Azienda Ospedale Università Padua Italy; ^5^ Ospedale San Bonifacio, Local Health Unit Scaligera Verona Italy; ^6^ Veneto Tumour Registry, Azienda Zero Padua Italy

**Keywords:** CIN2, HPV genotyping, methylation, p16/ki67, regression

## Abstract

Cervical intraepithelial neoplasia grade 2 (CIN2) can spontaneously regress in a sizable proportion of cases. The aim of this prospective multicenter cohort study was to identify the biomarkers associated with a high probability of regression. A total of 319 women aged 25–45 years fulfilling predefined inclusion and exclusion criteria (full visibility of transformation zone and lesion; no previous history of CIN2+ or immune impairment) were enrolled and subjected to active surveillance for 24 months. HPV genotyping, p16/ki67 expression, methylation status of *FAM19A4/miR124‐2* genes, and cytology were evaluated at baseline. The probability of CIN2 regression according to the different biomarkers was evaluated through binomial logistic regression. At follow‐up, regression, persistence, and progression (evaluated on 294 women) were recorded in 165 (56%), 68 (23%), and 61 (21%) cases, respectively; no association with age was observed. Overall, 110 women underwent excisional treatment during follow‐up; 53 CIN2 and 50 CIN3+ were diagnosed. The probability of CIN2 regression significantly increased with early HPV negativity (odds ratio [OR] 6.45, 95% confidence intervals [CI] 1.68–42.6), no p16/ki67 expression (OR 2.49, 95%CI 1.38–4.52), and unmethylated status (OR 2.12, 95%CI 1.09–4.20). Our results indicate that active CIN2 surveillance could be implemented for women up to 45 years, after selection according to anatomo‐clinical criteria and biomarker status. To improve feasibility, the biomarkers can be used sequentially, taking advantage of the HPV genotyping available in primary screening tests, and eventually refining the selection by using the other biomarkers in selected subgroups.

## Graphical abstract

What's new?

Cervical intraepithelial neoplasia grade 2 (CIN2) lesions can spontaneously regress, especially in women aged under 30. Better predictive biomarkers are needed to improve risk stratification for active surveillance. This prospective multicenter cohort study evaluated HPV genotyping, p16/ki67 expression, and FAM19A4/miR124‐2 methylation status in cervical samples from women aged 25–45 years with CIN2 lesions managed conservatively. Regression—observed in 56% of the cases—was strongly associated with HPV negativity shortly after diagnosis, positivity for high‐risk HPV types different from 16/18, no p16/ki67 expression, and unmethylated status. These biomarkers could potentially be used to select CIN2 cases for conservative management.

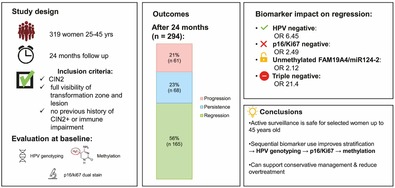

**Abbreviations**
AGC: atypical glandular cells
ASC‐H: atypical squamous cells cannot exclude high‐grade
ASC‐US: atypical squamous cells of undetermined significance
CIN: cervical intraepithelial neoplasia
Ct: cycle threshold
FIGO: International Federation of Gynecology and Obstetrics
hrHPV: high‐risk human papillomavirus
HR HPV non‐16/18: hrHPV other than 16/18
HSIL: high‐grade squamous intraepithelial lesion
LHU: Local Health Unit
LLETZ: large loop excision of the transformation zone
LSIL: low‐grade intraepithelial lesion
ODs: odds ratio
TZ: transformation zone

## 1 INTRODUCTION

Cervical intraepithelial neoplasia grade 2 (CIN2) lesions have a substantial probability (40%–60%, depending on age) of spontaneous regression,^1^, ^2^, ^3^, ^4^ and active surveillance can be applied instead of immediate treatment. The rationale for this conservative management is the possible adverse effects on subsequent pregnancies (i.e., preterm birth) caused by excisional procedures.^5^ So far, active surveillance has been mostly used in women of childbearing age (particularly if younger than 30), who benefit more than older women,^2^ as some studies have demonstrated higher regression rates in women aged <30.^4^

Some countries have developed specific guidelines,^3^ but conservative management is also performed in countries that do not have specific protocols.^6^ Since progression usually occurs within 2 years from the CIN2 diagnosis, active surveillance usually lasts 24 months, with semi‐annual follow‐up examinations.

The main concern in delaying CIN2 treatment is the risk of missing higher‐grade lesions that, if left untreated, could potentially evolve into carcinoma; in a recent study, active surveillance has been associated with a 4‐fold higher risk of cancer in the longer term.^7^ In order to minimize this risk, it is essential that the women at higher risk of progression are selected effectively. Selection criteria have so far included age, anatomo‐clinical features, and willingness to comply with follow‐up examinations.^8^ To improve the effectiveness of risk stratification, different biomarkers are under investigation.^9^ These include HPV genotyping,^10^ p16/ki67 overexpression, and methylation status of cellular and/or viral sequences.^11^, ^12^, ^13^

In Italy, there has been organized population‐based cervical cancer screening since 1996, and HPV primary testing in women over 30 since 2014 (regional programmes). There are ongoing investigations into strategies to increase screening efficiency by improving risk‐based approaches; in particular, there are studies on the use of triage biomarkers other than cytology^14^ and on more personalized management strategies. A survey carried out in 2019 by the Italian scientific association GISCi (Italian Group for Cervical Cancer Screening) showed that alternative management for CIN2 in young women is being offered, both outside^15^ and as part of organized cervical cancer screening, despite the fact that Italian guidelines still recommend excision of all CIN2 and CIN3 lesions. Among the 249 healthcare professionals who responded to the questionnaire, the rate of CIN2 lesions not undergoing immediate treatment has increased over time (from 5% in 2006–2009 to 9% in 2011–2015), and the gynaecologists offering conservative management based their decision mainly on the woman's age and colposcopy results (data not shown, presented at the 2019 GISCi National Meeting, accessible at: https://gisci.it/documenti/convegni/riccione2019/20190531/9_Burroni_Garutti_Tinacci_gisci_2019.pdf).

Our prospective study, performed as part of the cervical cancer screening programmes in the Veneto region, aimed at evaluating the clinical outcome of CIN2 lesions managed conservatively and the prognostic value of different biomarkers (HPV genotyping, p16/ki67 dual stain, cellular methylation) tested in the cervical cells for predicting regression. This paper presents the rates and times of the clinical outcomes over 24 months, in relation to the baseline results of the biomarkers, considered either alone or in combination.

## 2 MATERIALS AND METHODS

### 2.1 Study population

All the women aged 25–64 years attending population‐based organized cervical cancer screening programmes during the period April 15, 2019 to October 31, 2021 with a diagnosis of CIN2 were evaluated for enrolment in the study, according to predefined criteria, as previously described.^16^ The primary test was cytology for those aged 25–29 and HPV test (high‐risk types only) for those aged 30–64. The inclusion criteria were: histologically confirmed diagnosis of CIN2 (original diagnosis was used); age 25–45 years; transformation zone (TZ) fully visible at colposcopy. The exclusion criteria were: existing pregnancy (women who became pregnant after enrolment into the study were allowed to remain part of the study); previous treatment of a CIN2+ lesion; immunodeficiency; presence of an endo‐cervical lesion not completely visible at colposcopy. Eligible women were provided with detailed information on the routine management of CIN2 lesions (natural history and the risks of the treatment in use) and the protocol of the study. Women accepting conservative management (i.e., active surveillance instead of immediate excisional treatment) were enrolled in the study after signing a consent form.

### 2.2 Study design and definition of clinical outcomes

A prospective multicenter study was carried out by the gynaecology clinics of the screening programmes located in Mestre‐Venezia, Padua, Treviso, and Verona (Veneto region, North‐East Italy). HPV‐based screening was implemented through pilot projects in Padua and Mestre‐Venezia in 2010/2011 (for women aged 25–64), and in 2015 in Treviso and Verona (following regional‐scale implementation). At baseline and at every follow‐up examination (scheduled at 6, 12, [18, optional in case of negative results at the 12 months visit] and 24 months), cytology, colposcopy,^17^ immunocytochemistry, and molecular analyses were performed, as previously described.^16^

Surgical treatment was scheduled in case of progression to CIN3+ and after CIN2 persistence for >12 months. Since part of the study was conducted during the COVID‐19 pandemic, this could have influenced some clinical decisions. At follow‐up visits, punch biopsies were taken at the discretion of the colposcopist only.

Clinical outcomes were defined as follows: the worst diagnosis within the 24‐month time period was considered, regarding histology as superior to cytology. Progression: histological diagnosis of ≥CIN3 (CIN3/AIS, carcinoma) and/or HSIL (high‐grade squamous intraepithelial lesion)/ASC‐H (atypical squamous cells cannot exclude high‐grade)/AGC (atypical glandular cells) cytology. Persistence: histological diagnosis of CIN2 (and/or LSIL cytology). Regression: histological diagnosis of ≤CIN1, and/or negative/ASC‐US cytology. Clinical information up to 24 months from enrolment was integrated by data from the local pathology databases on additional cytology and/or histology exams performed outside the screening facilities.

### 2.3 Specimen characteristics and processing

Clinician‐taken cervical samples were collected in PreservCyt solution; the baseline sampling occurred at the visit generally scheduled 2 months after the routine colposcopy for informing the woman of its results. DNA extraction was performed using the QIAmp DNA Mini Kit (Qiagen, Hilden, Germany), followed by quantification with the Qubit 2.0 fluorimeter (Life Technologies), as previously described.^18^ In all cases where the amount of DNA was less than 200 ng, the QIAmp DNA Micro Kit (Qiagen, Hilden, Germany) was used. Since the quantity of DNA of some samples was still not adequate for subsequent analyses, changes were made to the extraction protocol to increase the yield; consequently, the sample volume was increased from 3 to 10 mL for samples with very low DNA yield.

The protocol and methodologies applied in the study are described in a paper previously published in the EJGO journal^18^; in this paper, only the subsequent modifications are described.

#### 2.3.1 Searching for and genotyping HPV sequences

All samples have been tested by Cobas 4800 HPV assay (Roche), that provides partial HPV16/18 genotyping. Samples positive for other HR types only were tested by PCR with MY09/MY11 primers, targeting the L1 region and detecting high‐ and low‐risk types, and the amplicons were analyzed by RFLP (restriction fragment length polymorphism) analysis to determine the specific HPV type. Positive and negative controls were included. DNA validity was assessed by PCR with the GH20/PC04 primers for the beta‐globin gene.

Samples untypable by the in‐house method were additionally tested using the commercial kit EasyPGX® ready HPV (Diatech Pharmacogenetics), a real‐time PCR assay amplifying the E6 and E7 oncogenes of 14 high‐risk HPV genotypes (16, 18, 31, 33, 35, 39, 45, 51, 52, 56, 58, 59, 66, and 68). The kit, used according to the manufacturer's instructions, detects the different HPV types in four multiplex reactions (FAM channel: types 16, 18, 33, 45; ROX channel: types 31, 51, 56, 58; Cy5 channel: types 39, 52, 35/59, 66/68; HEX channel: internal control). Water is used as a negative control and a synthetic mixture of DNA sequences positive for all the detectable types as a positive control. Co‐amplification of the human β‐actin gene serves as an internal control.

The extracted DNA is diluted to obtain a final volume of 25 μL containing 25–50 ng, which is added to each of the four mixes. DNA amplification is performed on an EasyPGX® instrument. The results are automatically analyzed using EasyPGX® Analysis software version 4.0.0 that evaluates the quality and quantity of DNA used in each sample and determines the HPV genotype for the valid samples, presenting the results in a table.

HPV types were classified hierarchically and grouped in two (for partial genotyping: 16/18, non‐16/18) or in three (for extended genotyping: 16/18, 31/33/35/45/52/58, and 39/51/56/59/66/68) groups, also in cases of multiple infections. Each woman was included in one group only.

#### 2.3.2 Methylation status of *FAM19A4* and *miR124‐2* cellular genes

DNA bisulfite conversion was performed using the EZ DNA Methylation Kit (Zymo Research), using 200 ng of genomic DNA, and eluting the modified DNA in 10 μL.

The QIAsure methylation assay (Qiagen) was used to analyze the methylation status of the promoters of the *FAM19A4* and *miR124‐2* genes. 2.5 μL of bisulfite‐converted DNA was used as a template in the PCR, performed on the Rotorgene PCR platform (Diatech Pharmacogenetics). The β‐actin gene served as a reference. The results were expressed as Ct (Cycle threshold) values, and interpreted according to the manufacturer's instructions. The housekeeping β‐actin (ACTB) gene was used as a reference to monitor bisulfite conversion and sample quality; DNA from HPV16‐positive SiHa cells was used in each run as a methylation‐positive control. A sample was considered positive if at least one of the two markers was positive. Invalid samples were retested using a new DNA extraction, and considered inadequate after two invalid results.

#### 2.3.3 p16/ki67 dual stain immunocytochemistry

Cytology slides for p16/ki67 immunocytochemistry were prepared using a T2000 slide processor and the CINtec® Plus kit (Roche). All slides were reviewed by an experienced cytotechnologist to detect the staining performance of the two markers and were considered positive with ≥1 cell stained for both p16 and ki67. A slide containing cells from a previously tested sample was concomitantly processed in each immunocytochemistry run for quality assurance. Slides with insufficient staining or with an insufficient number of cells were considered inadequate.

#### 2.3.4 Cytology

Liquid‐based slides (ThinPrep, Hologic) were prepared and interpreted in local screening pathology laboratories using the Bethesda 2001 classification.^19^

### 2.4 Statistical analysis

We described the frequency of all the study biomarkers (overall and by clinical outcome—regression/persistence/progression), of the clinical outcomes, and of the surgical interventions performed during the study. The clinical outcome was dichotomized as progression or persistence versus regression. The chi‐square test and the Fisher's exact test were initially used to evaluate the proportion of clinical outcomes for study biomarkers. A binomial logistic regression model was then performed including partial and extended genotyping, p16/ki67 dual stain, cellular methylation, and a combination of these as independent variables, and progression/persistence as the dependent variable.

The frequency of regression was also evaluated in relation to the screening history of the women by comparing women at first versus women at subsequent HPV‐based screening round.

*p*‐values less than 0.05 were considered statistically significant. Statistical analyses were performed using the R software, version 4.2.1 (R Core Team, 2022). No adjustment for potential confounders was performed.

## 3 RESULTS

### 3.1 Study population, follow‐up data, and clinical outcome

A flow chart with the selection of women for this study has been published in a previous paper.^16^ In brief, out of 640 CIN2 cases detected by the study centers during the enrollment period, 228 (35.6%) women were excluded according to the predefined criteria and 412 (64.4%) were eligible. Among the latter, 93 women refused to enter the study and 319 accepted. Out of the 319 women aged 25–45 years (median age 33 years) with a histological diagnosis of CIN2 enrolled in the study from April 2019 to October 2021, only the results for the 294 with evaluable follow‐up are presented. A summary of the relevant anatomo‐clinical characteristics (age, index cytology, colposcopy findings) and the biomarker results at baseline is reported in Table 1. After enrollment, 15 women (5%) became pregnant, and all but one remained in the study.

**TABLE 1:** Main characteristics at baseline of the 294 women of the study with evaluable data.

	*n* (%)
Age (years)	
25–29	85 (28.9%)
30–34	96 (32.6%)
35–39	66 (22.5%)
40–45	47 (16.0%)
Cytology	
Negative	29 (9.9%)
ASC‐US	16 (5.5%)
LSIL	113 (38.4%)
HSIL	76 (25.8%)
ASC‐H	43 (14.6%)
AGC	2 (0.7%)
na	15 (5.1%)
Colposcopy	
Negative	15 (5.1%)
G0	8 (2.7%)
G1	181 (61.6%)
G2	89 (30.3%)
na	1 (0.3%)
HPV partial genotyping	
Negative	26 (8.8%)
HPV 16/18	127 (43.2%)
HR HPV (non‐16/18)	141 (48.0%)
HPV extended genotyping	
Negative	26 (8.8%)
HPV 16/18	127 (43.2%)
HPV 31/33/35/45/52/58	119 (40.5%)
HPV 39/51/56/59/66/68	16 (5.5%)
nd	6 (2.0%)
p16/ki67 dual stain	
Negative	159 (54.1%)
Positive	117 (39.8%)
na	18 (6.1%)
FAM19A4/miR124‐2 methylation	
Negative	210 (71.4%)
Positive	69 (23.5%)
na	15 (5.1%)

Abbreviations: ASC‐US, atypical squamous cells of undetermined significance; LSIL, low‐grade intraepithelial lesion; HSIL, high‐grade squamous intraepithelial lesion; ASC‐H, atypical squamous cells cannot exclude high‐grade; AGC, atypical glandular cells; G0, normal; G1, minor changes; G2, major changes; na, not available or invalid result; nd, HPV genotype not determined.

**FIGURE 1:**
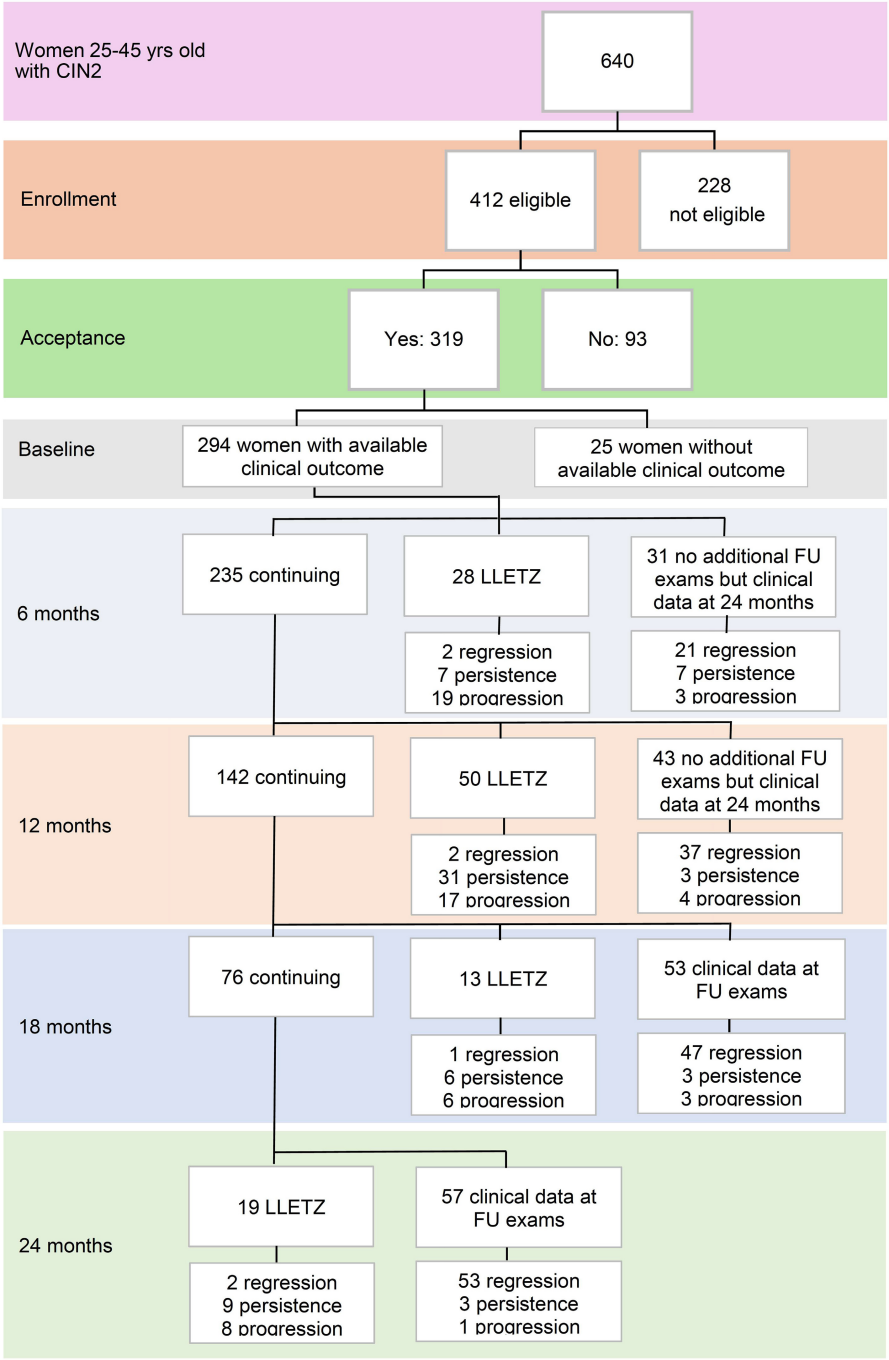
Flow chart of follow‐up examinations and outcomes for 294 women with a CIN2 diagnosis enrolled in a prospective study on conservative management.

Compliance with follow‐up examinations up to 18–24 months was recorded for 142 (44.5%) women. Of the remaining 177 women, 78 underwent out‐of‐protocol excisional treatment (28 after the baseline visit and 50 during follow up); for a further 74, clinical data up to 24 months from enrollment, which allowed us to establish the clinical outcome, was retrieved (as outlined in Figure 1); while 25 (7.8%) were lost.

Among the 294 women with established outcomes, regression occurred in 165 (56.1%), persistence in 68 (23.1%), and progression in 61 (20.8%). Pathologic confirmation was available for 7/165 women with regression (4.2%), 53/68 with persistence (77.9%), and 50/61 with progression (82%). Lesion progression occurred mostly within 12 months (43/61, 70%), while regression increased over time.

By comparing women at their first HPV test at study entry with those at subsequent HPV‐based screening rounds, we observed statistically significant (*p* = .0035) different regression rates, that is, 48% (109/229) and 58% (52/90), respectively.

Overall, during the study period, excisional treatment was performed in 110/294 (37.4%) women; ≥CIN3 was diagnosed in 50 cases (43 CIN3, 5 AIS, 1 micro‐invasive carcinoma and 1 invasive carcinoma, FIGO stage IA1), CIN2 in 53, and CIN1 or less in 7. The number of excisional treatments during follow‐up varied by calendar period, with an excess at the 12‐month examination during the COVID‐19 lockdown.

### 3.2 Biomarker data

All the biomarkers were evaluated in the cervical cell samples obtained at study entry (i.e., a median of 2 months after the CIN2 index biopsy) from all the enrolled women. Partial genotyping was determined for all samples, while extended genotyping could not be determined for six samples, although we used three different methods to define the HPV type(s) present; the very weak signal observed is compatible with a low viral load, and the results of all the assays could not be interpreted with certainty. Invalid/missing data were recorded for nearly 6% for p16/ki67 and for nearly 5% for methylation analyses.

The results of the HPV genotyping are reported in Table 1, divided by partial and extended genotyping. The types are listed hierarchically as HPV16/18 (single and mixed infections) and HR HPV (non‐16/18) for partial genotyping, and grouped according to the oncogenic risk as HPV16/18 (high), HPV 31/33/35/45/52/58 (intermediate), HPV 39/51/56/59/66/68 (low) for extended genotyping.

Overexpression of p16/ki67 proteins was observed in 117 cases (39.8%); 159 cases were negative, and 18 were invalid.

Hypermethylation status of the cellular genes *FAM19A4/miR124‐2* was recorded in 69 cases (23.5%), of which 11 were for both genes, 5 were for *FAM19A4* only, and 53 were for *miR124‐2* only; 210 cases were negative and 15 were invalid.

### 3.3 Clinical outcomes according to biomarkers' results at the baseline

**FIGURE 2:**
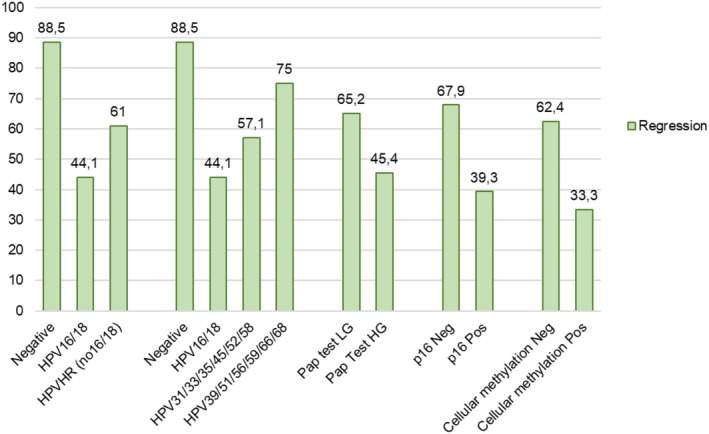
Regression within 24 months of enrolment among 294 women with CIN2 conservatively managed, by biomarker results at baseline.

The clinical outcomes (regression, persistence, or progression) according to baseline biomarkers (alone or in combination) are reported in Table 2 and Figure 2. Regression was significantly associated with HPV types other than 16/18 (109/165, 66.1%), low‐grade cytology (103/158, 65.2%), no expression of p16/ki67 proteins (108/165, 65.4%), and negative methylation status (131/165, 79.4%). On the other hand, progression was associated with HPV types 16/18 (35/61, 57.4%) and p16/ki67 expression (40/61, 65.6%).

**TABLE 2:** Distribution of clinical outcomes by biomarker results at baseline for women with available follow‐up data and valid result for the corresponding biomarker.

Total	Regression	Persistence	Progression	*p*‐value^a^
	*N* (%)	*N* (%)	*N* (%)	
	165 (56.1%)	68 (23.1%)	61 (20.8%)	
HPV partial genotyping				<.001
Negative	23 (88.5%)	1 (3.8%)	2 (7.7%)	
16/18	56 (44.1%)	36 (28.3%)	35 (27.6%)	
HR HPV (non‐16/18)	86 (61.0%)	31 (22.0%)	24 (17.0%)	
HPV extended genotyping				<.001
Negative	23 (88.5%)	1 (3.8%)	2 (7.7%)	
16/18	56 (44.1%)	36 (28.3%)	35 (27.6%)	
31/33/35/45/52/58	68 (57.1%)	30 (25.2%)	21 (17.6%)	
39/51/56/59/66/68	12 (75.0%)	1 (6.25%)	3 (18.75%)	
Cytology^b^				.002
Low grade	103 (65.2%)	32 (20.2%)	23 (14.6%)	
High grade	55 (45.4%)	32 (26.5%)	34 (28.1%)	
p16/ki67 dual stain				<.001
Negative	108 (67.9%)	32 (20.1%)	19 (12.0%)	
Positive	46 (39.3%)	31 (26.5%)	40 (34.2%)	
Methylation				<.001
Negative	131 (62.4%)	44 (20.9%)	35 (16.7%)	
Positive	23 (33.3%)	24 (34.8%)	22 (31.9%)	

^a^ The *p*‐value refers to differences in the proportion of outcomes by biomarkers' value at baseline. *p*‐values according to univariate analysis.

^b^ Low grade includes ASC‐US/LSIL; high grade includes ASC‐H/HSIL/AGC.

The univariate analysis was performed on biomarkers by testing different categories of variables. The most significant categories, on which the multivariable analysis was conducted by comparing regression to persistence and progression combined, are reported in Table 2.

The odds of regression by biomarkers (alone or in different combinations) are summarized in Table 3. A statistically significant association with regression was found for negative p16/ki67 (OR 2.49, 95%CI 1.38–4.52) and unmethylated status (OR 2.12, 95%CI 1.09–4.20) considered as a single biomarker. Combining the three variables (HPV type, p16/ki67 and methylation status), the ORs in all cases of negativity for both p16/ki67 and methylation, irrespective of the HPV typing result, were six to eight times higher than in patients with HPV 16/18, positive p16/ki67, and the presence of methylation. A significant association with regression was also found in the case of HR HPV non‐16/18, positive p16/ki67, and unmethylated status (OR 3.90, 95%CI 1.24–13.2).

**TABLE 3:** Odds ratios for regression by single biomarker and by combination of HPV genotyping, p16/ki67 dual stain, and methylation status, with 95% confidence intervals and *p*‐value, according to multivariate analysis.

Biomarker			Number of patients	OR	95%CI	*p*‐value
p16/ki67 dual stain						
Positive (reference)			117	1.00		
Negative			159	2.49	1.38–4.52	.003
Cellular methylation						
Positive (reference)			69	1.00		
Negative			15	2.12	1.09–4.20	.028
Cytology						
High grade			121	1.00		
Low grade			129	1.71	0.97–3.01	.064
HPV genotyping						
16/18			127	1.00		
HR HPV (non‐16/18)			141	1.29	0.72–2.30	.4
Negative			26	6.45	1.68–42.6	.017
Combination of HPV genotyping, p16/ki67 dual stain, and methylation						
Genot.^a^	p16/ki67^b^	Methyl.^c^				
16/18	POS	POS	32	1.00		
16/18	POS	NEG	35	2.11	0.73–6.51	.2
16/18	NEG	POS	12	3.57	0.88–15.2	.076
16/18	NEG	NEG	42	6.43	2.34–19.5	<.001
HR^d^	POS	POS	16	1.62	0.40–6.28	.5
HR^d^	POS	NEG	23	3.90	1.24–13.2	.023
HR^d^	NEG	POS	6	3.57	0.56–23.5	.2
HR^d^	NEG	NEG	71	7.95	3.13–22.5	<.001
NEG	NEG	NEG	21	21.4	5.47–113	<.001

^a^ HPV genotyping.

^b^ p16/ki67 dual stain.

^c^ Methylation.

^d^ HR HPV (non‐16/18).

## 4 DISCUSSION

In this multicenter prospective cohort study, nested within organized cervical cancer screening, 319 women aged 25–45 with a diagnosis of CIN2 lesion were managed by active surveillance for up to 24 months. Among the 294 women with evaluable follow‐up, the observed regression, persistence, and progression rates were 56%, 23%, and 21%, respectively. These figures are in line with other studies,^4^, ^8^, ^11^, ^20^ and confirm the occurrence of spontaneous CIN2 regression in a sizable proportion of women.

The results of HPV genotyping (high‐risk types), p16/ki67 dual stain, and *FAM19A4/miR124‐2* methylation assays were evaluated as potential predictive biomarkers. The strongest association with CIN2 regression was observed for: HPV negativity shortly (median of 2 months) after CIN2 diagnosis; positivity for high‐risk HPV types different from 16/18 (with highest figures for the low oncogenic types 39, 51, 56, 59, 66, 68); no expression of p16/ki67 proteins, and unmethylated status. The association was statistically significant when the biomarkers were evaluated both individually and in combination. Comparisons were carried out between regression and the other outcomes individually, and between regression and persistence/progression combined; the results were comparable, thus we chose to present the data referring to the analyses for regression versus persistence/progression. On the other hand, the risk of progression was highest for women with HPV16 or HPV18, p16/ki67 overexpression, and hypermethylation of the cellular genes *FAM19A4/miR124‐2*.

In particular, the rate of regression was particularly high among patients without p16/ki67 protein expression alone (regression in 67.9% cases), or in combination with non‐16/18 types (65.3%), or with HPV16/18 (57.4%) when unmethylated status was also present.

The correlation between HPV type and clinical outcome has been previously reported,^10^, ^12^, ^21^ mainly for HPV16 versus non‐16‐HPV types. In our study, this association was significant also for HPV16/18 versus HPV non‐16/18 (as seen also by Damgaard et al.^21^). Moreover, by grouping HPV non‐16/18 types according to their oncogenic potential, we showed a strong correlation between regression and the low oncogenic types 39, 51, 56, 59, 66, 68 (75% regression rate).

The correlation between the methylation status of the *FAM19A4/miR124‐2* cellular genes and the clinical outcome was significant when considered alone but dominated by both HPV type and p16/ki67 status when considered in combination. Similar data have been found among the women enrolled in the CONCERVE study; the unmethylated status of *FAM19A4/miR124‐2* genes was predictive of regression in cases with low‐grade cytology but not in cases with high‐grade cytology and allowed the identification of women with very high regression incidence when combined with the absence of HPV16.^22^

According to our protocol, women up to 45 years were eligible for conservative management, and no association between age and clinical outcome was observed, in line with other studies.^12^, ^22^ While the majority of published studies consider an upper age limit of 30 years,^8^ in Denmark a conservative management for CIN2 can be offered to all women of reproductive age and recommended to those with a childbearing desire.^4^ Since the women who benefit the most from avoiding excisional treatment are those of childbearing age, and in many countries, the first pregnancy often occurs beyond the age of 30, the upper age limit is an important issue. Indeed, the use of biomarkers capable of selecting the women with the highest probability of regression can improve the safety of active surveillance and allow the eligibility age range to be extended. Moreover, since in our study the acceptance rate among the eligible women was above 77%, with insignificant differences by woman's age,^16^ acceptance of active surveillance appears to be independent of age.

Among the biomarkers evaluated in our study, besides differences in their predictability of clinical outcome, laboratory issues and routine applicability must also be considered. Within HPV‐based screening, partial and extended genotyping are already (or becoming increasingly) available with the primary clinically validated assays.^23^ On the other hand, both the p16/ki67 dual stain and methylation assays will incur additional costs that must be weighed against the cost reductions (primarily from not performing the surgical procedure).

For HPV‐based screening, cytology is the most frequently used assay to decide on the subsequent management for HPV‐positive women. Among the women included in our study, the vast majority had an abnormal index cytology, with 44% classified as low‐grade (i.e., ASC‐US, LSIL) and 41% as high‐grade (i.e., HGSIL, ASC‐H, AGC). These figures are quite similar to those described among Danish women with CIN2 managed conservatively (46.4% and 48.8%, respectively), where eligibility criteria are non‐restrictive and all women of reproductive age are eligible.^12^

A delayed surgical procedure was performed during the 24‐month surveillance period in 37.4% (110/294) of the women, a figure close to the 36.1% observed in Denmark.^12^ In our study, the distribution over time of these procedures showed an excess after the 12‐month follow‐up examination for the women enrolled in 2019–2020. Since for most of these cases the diagnosis was CIN2, this finding suggests a negative influence of the COVID‐19 pandemic, which may have made the women and healthcare professionals more anxious, leading to overtreatment and an overestimation of the diagnosis of persistence.

Some strengths can be attributed to our study. We analyzed the association with CIN2 regression of three different biomarkers, besides cytology, to be considered alone or in combination. Second, the high acceptance rate of the study protocol by the women attending the population‐based cervical cancer screening program (77%) makes our cohort a good representative of the general population. Third, the capacity to retrieve follow‐up data in most women who failed to attend all the follow‐up examinations enabled the evaluation of the clinical outcome in 92% (294/319) of the enrolled women.

We also acknowledge some limitations. First, the small sample size of some groups, particularly those defined by combinations of different biomarkers, limits the statistical power for the evaluation of the association with the outcome of interest. Second, no expert revision of the histological diagnoses was performed; we used the diagnoses of the local pathologists, who used p16 immunohistochemistry to resolve uncertain morphologies, a practice shown to be adequate and effective to ensure good reproducibility.^24^

## 5 CONCLUSIONS

On the basis of the results of our study, women with a CIN2 diagnosis, not previously treated for CIN2+, without immunodeficiency and with full visibility of the transformation zone and of the lesion, can be eligible for a conservative management up to age 45. A triage to select those with the highest probability of regression is feasible and advisable. To assure feasibility and sustainability, different triage strategies for the implementation of a CIN2 conservative management can be hypothesized, and the parameters can be used to discuss the conservative option with the woman, on the basis of her specific progression risk:

1. HPV testing with genotyping as a single biomarker: women screened by cytology with negative hrHPV early after diagnosis and women positive for non‐16/18 types only can be managed conservatively.
2. p16/ki67 dual stain as a single biomarker: women with a negative result can be managed conservatively.
3. *FAM19A4/miR124‐2* methylation status as a single biomarker: women with a negative result can be managed conservatively.
4. Strategies with combined biomarkers might select women with a very high probability of CIN2 regression (i.e., women positive for non‐16/18 types with negative p16/ki67 dual stain and/or unmethylated status), but additional data on larger groups are necessary.

## AUTHOR CONTRIBUTIONS

**Helena Frayle:** Methodology; investigation; writing – review and editing. **Silvia Gori:** Methodology; investigation; formal analysis; writing – review and editing. **Alessio Pagan:** Investigation; writing – review and editing. **Marika Soldà:** Investigation; supervision; writing – review and editing. **Cesare Romagnolo:** Investigation; writing – review and editing. **Egle Insacco:** Investigation; writing – review and editing. **Licia Laurino:** Investigation; writing – review and editing. **Mario Matteucci:** Data curation; writing – review and editing; software. **Giuseppe Sordi:** Writing – review and editing; investigation. **Enrico Busato:** Investigation; writing – review and editing. **Manuel Zorzi:** Conceptualization; supervision; writing – original draft; writing – review and editing. **Tiziano Maggino:** Conceptualization; funding acquisition; writing – review and editing; project administration. **Annarosa Del Mistro:** Conceptualization; supervision; validation; writing – original draft; writing – review and editing.

## CONFLICT OF INTEREST STATEMENT

The authors declare no conflicts of interest.

## ETHICS STATEMENT

Approval for the study was obtained from the Ethics Committee of the province of Venice and San Camillo Hospital (Venice, Italy, ethics ID 79A/CESC, 07/11/2017). Additional approvals were obtained from the other participating centers (Ethics Committee of the provinces of Treviso and Belluno, ethics ID 595/CE Marca, 22/11/2018; Ethics Committee of the provinces of Verona and Rovigo, ethics ID 2062/CESC, 19/02/2019; Ethics Committee of the province of Padua, ethics ID 4723/AO/19, 27/06/2019). Written informed consent was obtained from each participant. The study was carried out in accordance with the Declaration of Helsinki. The study has been registered in ClinicalTrials.gov (ID: NCT04687267).

## Data Availability

The de‐identified data that support the findings of this study are available from the corresponding author upon reasonable request and after approval by the Ethics Committee.
